# Post-Traumatic Stress Disorder Delineating the Progression and Underlying Mechanisms Following Blast Traumatic Brain Injury

**Published:** 2018-03-02

**Authors:** Brandon Lucke-Wold, Richard Nolan, Divine Nwafor, Linda Nguyen, Cletus Cheyuo, Ryan Turner, Charles Rosen, Robert Marsh

**Affiliations:** 1 Department of Neurosurgery, West Virginia University School of Medicine, Morgantown, WV, USA; 2 Center for Neuroscience, West Virginia University Health Science Center, Morgantown, WV, USA; 3 Department of Pediatric Neurology, University of California San Diego, San Diego, CA, USA

**Keywords:** Posttraumatic stress disorder, Blast traumatic brain injury, Hypothalamic pituitary axis, Novel treatments, Molecular mechanisms

## Abstract

Posttraumatic Stress Disorder (PTSD) is a devastating condition that can develop after blast Traumatic Brain Injury (TBI). Ongoing work has been performed to understand how PTSD develops after injury. In this review, we highlight how PTSD affects individuals, discuss what is known about the physiologic changes to the hypothalamic pituitary axis and neurotransmitter pathways, and present an overview of genetic components that may predispose individuals to developing PTSD. We then provide an overview of current treatment strategies to treat PTSD in veterans and present new strategies that may be useful going forward. The need for further clinical and pre-clinical studies is imperative to improve diagnosis, treatment, and management for patients that develop PTSD following blast TBI.

## Introduction

Posttraumatic Stress Disorder (PTSD) is categorized as both a trauma and stress related disorder and may present with a multitude of clinically significant symptoms lasting for at least a month. These symptoms are a direct result of exposure to a traumatic event such as the threat of death, actual death, serious injury, or sexual violence [1]. This disorder can affect any demographic, but there is a higher frequency of PTSD associated with the female sex, young age, unmarried status, and low household income, high levels of trauma exposure, high childhood adversity, having low self-esteem, and having a neurotic personality [2]. Symptom onset usually occurs within 3 months following the trauma, though there may be a significant delay from months to years, termed “delayed expression” [3]. A primary criterion for PTSD diagnosis is the presence of intrusion symptoms. These symptoms can include involuntary and distressing memories, dreams and flashbacks of the event, as well as any detrimental psychological and/or physiological reactions to cues that remind the patient of the traumatic event [3]. Obstinate avoidance of internal (memories, thoughts, feelings, etc.) and external (people, places, etc.) stimuli is another criterion important to the PTSD diagnosis [1]. Negative associations in cognition and mood related to the traumatic event are another hallmark feature of PTSD. These negative associations can include the inability to fully remember the event, exaggerated negative beliefs regarding oneself or others, blaming oneself or others for the event or its consequences, diminished interest in activities, a constant negative emotional state and/or inability to experience positive emotions, and feelings of social detachment [4]. Altered states of arousal are also important for the diagnosis of PTSD and may include irritable/angry behavior, an exaggerated startle response, reckless behavior, hyper vigilance, problems with concentration, and sleep disturbances [5]. PTSD may also present with or without dissociative symptoms, such as depersonalization and derealization [6]. The severity of PTSD symptoms is inversely related to mental and physical functional capabilities [7]. It has also been shown that those suffering from PTSD are at an increased risk of committing violent crimes against their intimate partner [8]. Unfortunately, this may propagate the problem by causing the partner to become inflicted with PTSD symptoms themselves, especially when coupled with violence-related traumatic brain injury (TBI) [9].

While most people will experience a traumatic event deemed sufficient to cause PTSD [10], there is less than a 10% lifetime PTSD prevalence among the general population [11]. PTSD disproportionally affects returning combat veterans compared to the general population [7]. According to the U.S. Department of Veterans Affairs, it is estimated that up to 30.9% of Vietnam veterans, 10.1% of Gulf War veterans [12], and 13.8% of Operation Enduring Freedom/Operation Iraqi Freedom have been afflicted with PTSD [13]. A potential underlying factor contributing to the relatively high rates of PTSD seen in veterans is the occurrence of a TBI. In the span of approximately 15 years (from January 1, 2000 to June 5, 2015) there have been over 327,000 cases of TBI and over 138,000 cases of PTSD among U.S. military personnel, many of which are comorbid [14,15]. It has been shown that mild TBI (mTBI) is strongly associated with PTSD [2], especially when the mTBI is blast-related or coupled with loss of consciousness [16,17]. Along with this association, the presence of TBI and/or PTSD is often difficult to ascertain due to the common comorbidity of the two conditions as well as their overlapping symptomology [14,18,15]. Fortunately, modern neuroimaging techniques (such as Single-Photon Emission Computed Tomography) show promise in helping to distinguish between the two conditions [14,18]. While the occurrence of TBI is one potential contributing factor, there has been significant research regarding the hypothalamic-pituitary-adrenal (HPA)-axis and its role in the development of PTSD.

### Underlying pathophysiology of PTSD

PTSD was previously thought to be the body’s natural response to a traumatic event, sharing a similar neurological response profile like stress. New research suggests however, that the adaptation response in PTSD does not reflect the specific changes you would see in a typical stress response profile [19]. A major pathway implicated in PSTD is the Hypothalamic Pituitary Adrenal Axis (HPA-axis) [19,20].

### Molecular mechanisms

The HPA-axis is an important component of the neuroendocrine system and is comprised of a set of interactions between the hypothalamus, the pituitary gland, and the adrenal glands. The effects of the HPA-axis are modulated by the effects on glucocorticoids [21,22]. The responsibility of this axis is to coordinate neural and endocrine signaling in response to perceived stress via signaling from the periventricular nucleus, which in turn stimulates the release of corticotrophin-releasing hormone, and subsequently adrenocorticotrophic hormone [21,23]. Adrenocorticotrophic hormone is released into the general circulation where it stimulates the production of the glucocorticoids from the adrenal cortex. The glucocorticoids bind to various glucocorticoid receptors, and exert effects throughout the body [21]. HPA-axis dysfunction is implicated in multiple mental health disorders, including PTSD [21]. One theory underlying HPA-axis dysfunction and PTSD is that common glucocorticoid receptor polymorphisms, N363S and *Bcl1*, have an effect on PTSD frequency. Unfortunately, no significant associations were observed between these glucocorticoid receptor polymorphisms and PTSD development [24]. Another study probed for associations between the FKBP5 gene, which helps to regulate glucocorticoid sensitivity, and PTSD frequency. It was found that a single nucleotide polymorphism (SNP) of the FKBP5 was significantly associated with increased lifetime PTSD rates [25]. The same study also examined the association between PTSD and CRHR1, a neurotransmitter involved in corticotrophin-releasing hormone activity. CRHR1 is also known to regulate HPA-axis function and is associated with the impact that a traumatic event exposure has on an individual [25]. Polymorphisms of this gene were shown to have significant association with increased PTSD rates among participants [25,26]. Another potential underlying factor of PTSD is the Apolipoprotein E (APOE) gene that has some effect on regulation of the HPA-axis [27,28]. Genetic analysis of the Apolipoprotein E (APOE) gene yielded conflicting results in that there was no significant association between APOE polymorphisms and PTSD frequency among all veteran participants [2]. In the non-Hispanic African American population however, those with APOE ε4 allele homozygotes who were exposed to high levels of combat showed significantly higher rates of PTSD with worsened symptom severity [29]. While genetic polymorphisms affecting the HPA-axis have a role in predicting PTSD frequency, there are a multitude of other genes that when mutated, will exert an effect on the likelihood of PTSD development. These genes include RGS2, COMT, CHRNA5, TNFα, DRD2, BDNF, ANK3, and ANKK1 (Table 1) [25,2,30–34].

### Adaptability of the HPA axis in stress

Upon exposure to an acute stressful stimulus, the hypothalamus secretes corticotrophin-releasing factor (CRF), vasopressin and other regulatory neuropeptides to the anterior pituitary causing the release of adrenocorticotrophic hormone (ACTH) [35]. ACTH travels to the adrenal gland, binds to its corresponding receptor on the adrenal cortex and influences the release of cortisol, a chemical-mediator well known to decrease stress. Simultaneously, there is a release of dose dependent catecholamine (Norepinephrine and Epinephrine), which results in a coordinated response from multiple organs preparing to respond to an acute stress. This dose dependent release is relative to the severity of the stressful stimuli [36]. When the stressor is removed, a negative feedback restores the molecules released in excess back to their homeostatic levels [37,38]. In chronic stress, sustained cortisol release acts tonically on the HPA-axis to decrease the release of cortisol via negative feedback inhibition [20].

### Neural alterations of the HPA axis in PTSD

#### Cortisol alterations

In order for cortisol to exert its effect on the body, it must be able to bind to glucocorticoid receptors. The glucocorticoid receptor in major depressive patients exhibits an attenuated response in the presence of cortisol [39,40]. In contrast, PTSD patients from studies conducted on combat veterans showed that these receptors appear to have a more sensitive response to steroids like cortisol [41]. When a dexamethasone suppression test was conducted on PTSD patients, Yehuda et al. found that strong receptor suppression occurred at the HPA-axis at low dexamethasone levels (0.5mg) compared to high levels of dexamethasone in PTSD patients [41]. This finding gives clarity to the presence of high CRF levels and low cortisol concentrations in PTSD due to the enhanced inhibitory feedback of cortisol at low levels because of the increased sensitivity of the glucocorticoid receptors [41,42].

#### Norepinephrine alterations

In humans, norepinephrine released from the locus ceruleus is involved in the regulation of mood, emotion and alertness (fight or flight) through increased peripheral sympathetic activity [43]. In the pathophysiology of PTSD, this sympathetic increase is exaggerated and observed as a high systolic and diastolic pressure with a concomitant increase in heart rate in PTSD veterans [44,45]. Studies have been conducted that measured the urinary catecholamine levels in patients with PTSD versus patients with other psychiatric conditions (Table 2). It was found that patients who had PTSD had higher levels of urinary catecholamine compared to patients with other psychiatric conditions or compared to patients who suffered a traumatic event without PSTD [46]. Another study that measured cerebrospinal fluid (CSF) norepinephrine levels came to the same conclusion. There was a rise in CSF norepinephrine levels when war-related PTSD veterans where exposed to combat themed videos, which correlated to worsening of mood in these veterans [47]. Consider other evidence from experiments conducted by pietrzak et al. showing that there was a decrease in the number of norepinephrine reuptake transporters in the locus ceruleus of PTSD patients compared to patients exposed to trauma without PTSD. These findings strengthen the proposed hypotheses of brain alterations in PTSD with changes in norepinephrine levels compared to normal patients [48,49].

#### Minor neurotransmitter alterations

Serotonin produced in the dorsal raphe nucleus is chronically low in PTSD and can lead to anxiety, impulsivity and aggressive-like behaviors. Another neurotransmitter thought to play a role is Dopamine but its role is not well understood because low levels of dopamine produce anhedonia, apathy and impaired attention seen in PTSD while high levels contribute to the agitation and restlessness [50].

### Physiologic and functional progression of PTSD

The theoretical mechanism of PTSD is not known but it is likely influenced by various factors that could potentially increase mortality and morbidity. Disruptive mechanisms of the HPA-axis and adrenal gland in PTSD have been implicated as a risk factor for the development of cardiovascular disease (CVD). Elevated catecholamine levels in PTSD simultaneously increase the risk of CVD due to its direct effect on the heart, blood vessels, and platelets. Consequentially this increase leads to an increased blood pressure and increased coagulation [51–53]. Chronic inflammation is also an important mechanism that is thought to play an important role in increased CVD risk of PSTD patients. Kanel et al. showed that the severity of PTSD symptoms was associated with the levels of increased inflammatory mediators like Tumor necrosis α and interleukin1β [54].

Wilson et al investigated whether oxidative stress and inflammation increase in the brain, adrenal gland and systemic circulation during the progression of PTSD in male rats exposed to predator cats. The results showed that PTSD rats experienced a diminished growth rate, increased adrenal gland weight and a decreased thymus weight after the 31-day experiment. There was also an increase in the total levels of reactive oxygen species (ROS) in the hippocampus, prefrontal cortex and adrenal glands of PTSD rats. Inflammatory mediators were also significantly increased in the brain, systemic circulation and adrenal gland [55]. These findings demonstrate a course of PTSD as a progressive systemic condition influenced by certain behavioral risk factors.

### Current treatment approaches

To date, there are no approved treatments for individuals with PTSD and TBI. Treatment for PTSD can be categorized as pharmacologic and non-pharmacologic, with treatment recommendation guidelines indicating stronger support for cognitive behavioral therapy (CBT) than medication interventions [56]. The greatest number of studies have been conducted on exposure-based treatments, with evidence supporting its use regardless of the type of trauma and comorbidities [57]. Exposure-based treatments involve having survivors repeatedly re-experience their traumatic event. Of the various approaches to exposure therapy, prolonged exposure (PE) has received the most attention [57]. PE aims toward fear extinction through both imaginal exposure (in which a patient repeatedly recounts memories of a trauma) and *in vivo* exposure (in which a patient is exposed to distressing situations in the present). Cognitive processing therapy (CPT), which focuses on challenging and modifying maladaptive beliefs related to trauma, is also widely supported in the treatment guidelines [56]. In addition to CBT, eye movement desensitization and reprocessing (EMDR) has been shown to be significantly effective, though the evidence base for EMDR has not been as strong as that for CBT [58]. Patients receiving EMDR engage in imaginal exposure to a trauma while simultaneously performing saccadic eye movements.

Despite their demonstrated efficacy in PTSD, only a few studies thus far have suggested that CBT may be an effective strategy to treat PTSD in patients with co-morbid PTSD and TBI. In a recent review of the literature on PTSD and TBI in 2014, Tanev et al. [59] suggests that PTSD treatments may be promising in individuals with co-morbid TBI, but the impact of TBI on the ability of patients with PTSD to benefit from the different forms of CBT, especially those with impaired cognition, remains to be elucidated. It would therefore be important to include a control group of subjects with PTSD but without TBI in future studies to examine first line CBT approaches in patients with co-morbid PTSD and TBI [59].

Among pharmacologic treatments, the strongest evidence exists for selective serotonin reuptake inhibitors (SSRIs). The only two FDA approved medications for PTSD are the SSRIs, sertraline and paroxetine. From the VA/DoD Clinical Practice Guidelines for PTSD, these SSRIs as well as fluoxetine (another SSRI) and venlafaxine (a serotonin norepinephrine reuptake inhibitor) are first-line recommended treatments based on large multi-site randomized clinical trials [60]. The guidelines also concluded that there is some benefit from and recommendations for the use of mirtazapine, prazosin (for nightmares/sleep), tricyclic antidepressants, nefazodone (with caution regarding liver failure) and monoamine oxidative inhibitors (with caution regarding drug-drug interactions and strict dietary controls) [60]. Compared to non-pharmacologic treatments, the evidence base for pharmacological treatments in co-morbid PTSD and TBI is even more limited. In the absence of randomized controlled trials, experts have recommend the following general principles [60] take a comprehensive approach, be aware that TBI is associated with a variety of other neuropsychiatric sequela; [61] obtain diagnostic clarity and initiate treatment trials with one agent at a time, with a clear diagnostic formulation (e.g., “I am treating TBI related cognitive deficits,” or “I am treating PTSD related sleep disturbance”); [62] begin with lower doses and use longer titration intervals because of heightened sensitivity to side-effects in patients with TBI; and [14] use longer treatment durations to assess efficacy because both TBI and PTSD are associated with heightened reactivity to environmental changes [63].

Of interest for future studies would also be whether the combination of medication and cognitive therapies is more effective than either treatment alone in patients with co-morbid PTSD and TBI. Based upon current knowledge, most prescribing clinicians view pharmacotherapy as an important adjunct to the evidenced based psychotherapies for PTSD rather than as mono therapy [64]. When using a combined approach of medication and therapy for PTSD, it is important to keep in mind the following practices: [60] coordination of care and treatment responses between therapist and clinician if they are separate entities [61] ongoing dialogue regarding medications and their side effects between clinician and patient; and [62] active patient role in his or her treatment [64]. These same practices should be applied to those with PTSD and TBI.

### Novel treatment strategies

As mentioned above, the mainstay of current PTSD treatment includes psychological therapy, CBT, and eye movement desensitization and reprocessing as well as use of antidepressant medications. However, only 20–30% of PTSD patients achieve complete remission, while the remaining 70–80% continues to be refractory to these treatment modalities [65]. Current investigations into novel therapies for treatment-refractory PTSD focus on modulation of the neuroanatomical, pathophysiological and molecular substrates of PTSD.

Central to the pathophysiology of PTSD is dysfunction of Pavlovian fear conditioning, characterized by impaired extinction and generalization of the conditioned fear. The amygdala and hippocampus have been implicated in the neurocircuitry of fear conditioning and fear generalization [66]. Neuro-functional imaging and lesional studies have shown that over-activity of the basolateral amygdala (BLn) is essential for the development of the clinical manifestation of PTSD [67]. These studies have established the basolateral amygdala as a specific target for modulation as a novel therapy for PTSD. Deep brain stimulation (DBS) is a treatment modality in which electrodes are stereotactically implanted into a specific brain target, which is then electrically stimulated to modulate its activity [68]. Studies have shown that rats traumatized by inescapable shocks, in the presence of a conspicuous object, had the tendency to bury the object when re-exposed to it 28 days later [69]. Using this pre-clinical model of PTSD, Langevin et al. found that rats treated with BLn DBS spent on average 13 times less time burying the ball than the sham control rats. The treated rats also spent 18 times more time exploring the ball than the sham control rats [70]. In a follow up study, Stidd et al. compared the effect of paroxetine (a serotonin selective inhibitor currently used to treat PTSD) with BLn DBS using the pre-clinical PTSD model. Paroxetine was found to decrease the measured general anxiety level of rats that underwent the PTSD protocol, but did not counteract shock-induced hyper-vigilance toward the trauma-associated object (ball). BLn DBS, however, did decrease shock-induced hyper-vigilance as measured by a lower burying time, but had no effect on general anxiety assessed in the elevated plus maze [71]. Based on these pre-clinical studies, a clinical trial is currently underway assessing basolateral amygdala DBS for treatment of PTSD in combat veterans (ClinicalTrials.gov Identifier: NCT02091843). The first case enrolled in this trial was a 48-year-old combat veteran with a baseline Clinician-Administered PTSD Scale (CAPS) score was 119, classifying him among the most severely ill patients. At 8 months after bilateral BLn DBS, the patient experienced a substantial clinical improvement and a 37.8% reduction in CAPS score from baseline [72]. The final results of this clinical trial will help determine the efficacy of BLn

### DBS for the treatment of PTSD

The hippocampus plays an essential role in memory formation and it is thought that hippocampal dysfunction may be associated with the impaired extinction and generalization of the conditioned fear seen in PTSD [73]. The hippocampus is unique in being one of two sites in the adult brain where neurogenesis occurs (the sub ventricular zone being the other site) [74]. Neurogenesis involves the proliferation of neural stem cells, their migration and differentiation into adult neurons. Studies have shown that loss of neural stem cells in the hippocampus is associated with clinical conditions of dementia such as Alzheimer’s disease [75]. Several studies have demonstrated that neurogenesis in the hippocampus is impaired in PTSD [61,76]. As mentioned above, generalization of fear is one of the central features of PTSD. Generalization is thought to result from failure of pattern separation, which is mediated by the hippocampus. Pattern separation occurs in the dentate gyrus (part of the hippocampal formation) when highly similar input firing patterns are coded into less similar output firing patterns within granule cell population of the dentate gyrus [77]. Neurogenesis is responsible for replacing worn out granule cells in the hippocampus. Thus, in clinical conditions associated with memory loss, there is loss of hippocampal granule cells, resulting in loss of hippocampal volume [78]. In a meta-analysis of studies that evaluated hippocampal volumes in PTSD, Ahmed-Letaio et al. found that PTSD patients had significantly reduced bilateral hippocampal volumes compared to healthy controls [62]. From the therapeutic standpoint, no treatment of PTSD targeting neurogenesis has been yet developed. Experimentally, neurogenesis may be harnessed for therapeutic purposes by promoting endogenous neurogenesis in the hippocampus or transplanting exogenous neural stem cells into the hippocampus. Modulation of endogenous neurogenesis in the hippocampus may be achieved by the use of biologics that stimulate the molecular pathways involved in neural stem cell proliferation, migration and differentiation. Using a rat model of PTSD, Nie et al. found that administration of rosmarinic acid (a component of Chinese herbal medicine) alleviated PTSD-like symptoms in rats exposed to an enhanced single prolonged stress paradigm and restored hippocampal proliferation and pERK1/2 expression. The effects of rosmarinic acid were inhibited by the blockage of the ERK signaling [79]. Further evidence of pERK1/2 modulation for the treatment of PTSD comes from the study by Peng et al., who demonstrated that administration of ziprasidone (atypical antipsychotic drug used for treating PTSD) reversed the anxiety-like behaviors in rats that exposed to an enhanced single prolonged stress paradigm, and also restored the proliferation and the protein expression of pERK1/2 and Bcl-2 in the hippocampus [80]. One of the main challenges to development of therapeutics for stimulating hippocampal neurogenesis is the impermeability of the blood brain barrier to most biologics. An alternative is the surgical transplantation of exogenous neural stem cells into the hippocampus. Wei et al. induced traumatic brain injury (TBI) and posttraumatic brain injury in Wistar rats. Three days after TBI, rats were treated with intracranial transplantation of either mouse iPSC-derived neural progenitor cells under normal culture conditions (N-iPSC-NPCs) or mouse iPSC-derived neural progenitor cells pretreated with hypoxic preconditioning (HP-iPSC-NPCs). They found that the HP-iPSC-NPC-transplanted animals showed a unique benefit of improved performance in social interaction, social novelty, and social transmission of food preference tests compared to vehicle, which was mediated by up regulation of social behavior-related genes, oxytocin and the oxytocin receptor [81]. The challenges to direct transplantation of neural stem cells for therapeutic purposes include non-survival of transplanted cells over the long-term and also the potential for malignant transformation. These challenges must be overcome through further research before neural stem cells can be harnessed for the treatment of PTSD.

Another area of active investigation for the development of novel therapeutics for PTSD is epigenetic modification such as DNA methylation and post-translational histone modifications. Important mediators of epigenetic modification include microRNAs (miR). Micro RNAs are single stranded, non-coding RNA short fragments with 19–24 nucleotides that function in RNA silencing and gene regulation by binding to complementary sequences on messenger RNA (mRNA) [82]. It is believed that 30% of human genes are regulated by miR and that 80% of miRs are tissue-specific. miR signatures of various psychiatric disorders have been characterized. Balakathiresan et al. evaluated miR expression in the serum and amygdala using a pre-clinical model of PTSD in rats. They found a panel of nine stress-responsive miRNAs, namely; miR-142-5p, miR-19b, miR-1928, miR-223-3p, miR-322*, miR-324, miR-421-3p and miR-463* and miR-674*, which may have potential as biomarker(s) for PTSD. Further analysis revealed five miRs, miR-142-5p, miR-19b, miR-1928, miR-223 and miR-421-3p, which may play a potential role in the regulation of genes associated with delayed and exaggerated fear [83]. Zhou et al. also analyzed the peripheral mononuclear cells and various lymphocyte subsets in combat veterans and found that the percentage of Th1 cells and Th17 cells increased, regulatory T cells (Tregs) decreased, while Th2 cells remained unaltered in PTSD patients. High-throughput analysis of mononuclear cells for 1163 miRs showed significant alteration in number of miRs and also revealed a relationship between selected miRNAs and genes that showed direct/indirect role in immunological signaling pathways [84]. Furthermore, Wingo et al. conducted genome-wide differential gene expression survey on patients with post-traumatic stress disorder (PTSD) with comorbid depression and found that blood DICER1 (a regulator of miR expression) levels were significantly reduced [85]. Taken together, these studies demonstrating a role of miR in the pathogenesis of PTSD, indicate that miR signatures in PTSD may represent potential therapeutic targets. However, further research is required in defining the exact role of miRs in PTSD before therapies can be developed. Currently, the only available miR-based therapeutic is miravirsen, which targets hepatitis C viral infection [86]. To develop similar treatments for PTSD, the significant challenge of blood-brain barrier permeability must be overcome.

**Figure 1:**
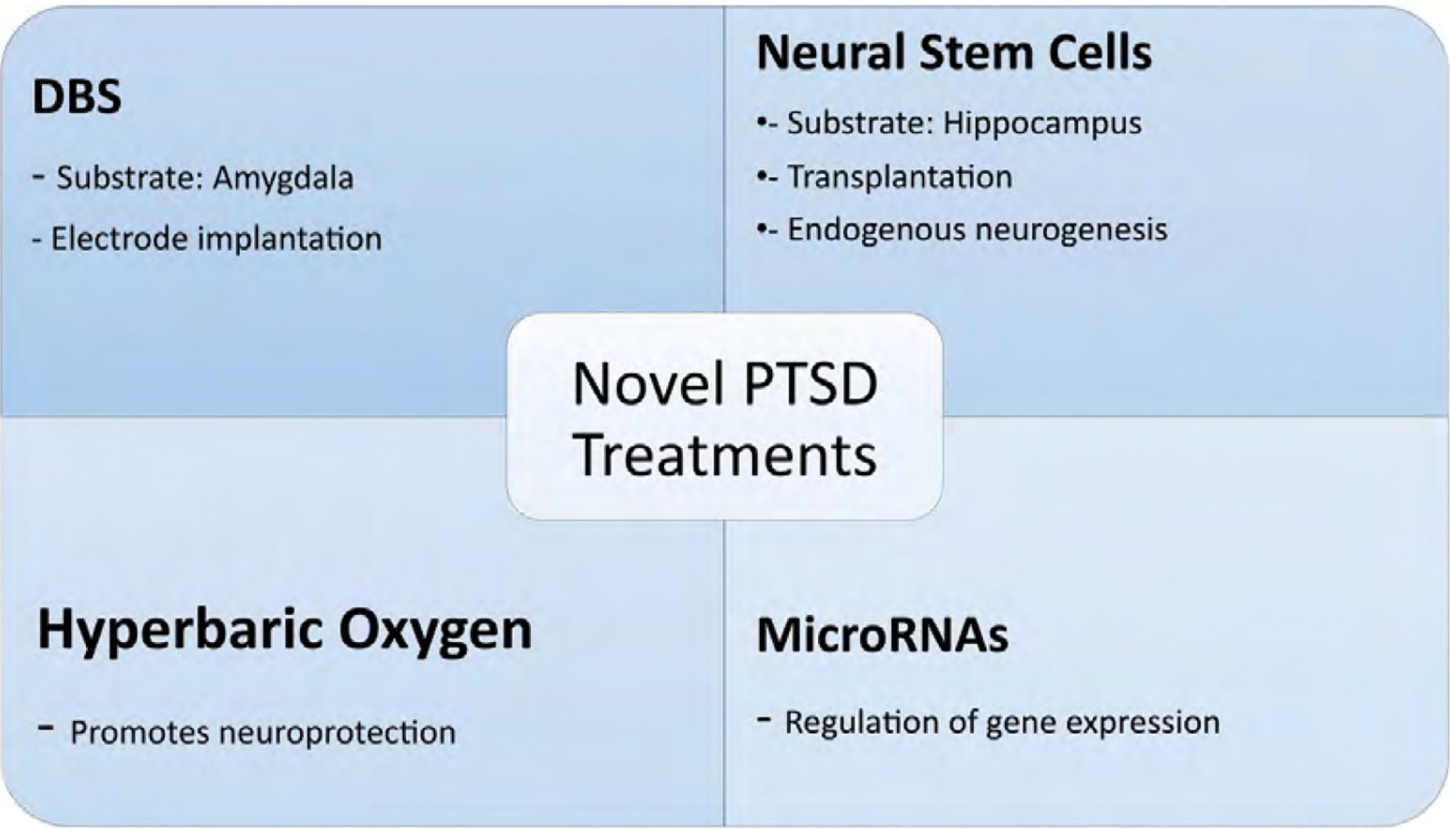
Novel PTSD treatments.

Another potential treatment modality for PTSD, which is currently under investigation, is hyperbaric oxygen therapy (HBOT). HBOT is defined as the delivery of 100% pressures greater than 1 Atmospheres Absolute while the patient is being pressurized in a chamber. HBOT is increasingly being used a field treatment modality in various military establishments such as NATO in Afghanistan and Iraq, where servicemen and women are frequently exposed to blast-induced traumatic brain injury and PTSD [87]. HBOT has been shown to mediate tissue healing via a variety of mechanisms including increasing oxygen delivery, stimulation of stem cell proliferation, reduction in apoptosis, up regulation of growth factors, production of antioxidant, and inhibition of inflammatory cytokines [88]. These molecular mechanisms of HBOT make it an attractive option for treatment PTSD as has been demonstrated in both pre-clinical and clinical studies. Peng et al. showed that hyperbaric oxygen preconditioning was able to significantly preserve viable neurons in the CA1 subfield of hippocampus in rats following single prolonged stress exposure, as evidenced by decreasing CA1 neuronal apoptosis. Furthermore, hyperbaric oxygen preconditioning was able to up regulate the expression of thioredoxin reductase and ameliorated anxiety-like behavior and cognitive impairments induced by the single prolonged stress [89]. Recently, a phase I clinical trial of HBOT was conducted among war veterans. The study demonstrated that HBOT resulted in significant improvement in symptoms, neurological exam, full-scale IQ, WMS IV Delayed Memory, WMS-IV Working Memory and quality of life, among other measures. Theses clinical improvements were associated with diffuse improvements in regional cerebral blood flow as measured by SPECT [90]. A meta-analysis of eight studies on HBOT showed that patients undergoing hyperbaric therapy achieved significant improvement in the GCS and GOS with a lower overall mortality [91]. The novel treatment modalities outlined above are summarized in Figure 1.

## Summary

PTSD continues to cause significant morbidity and mortality and the incidence of the disorder is expected to increase as more and more servicemen and women return home from war duties. Thus, the need for developing novel therapies for combating this clinical condition cannot be overemphasized. Current study has focused on the HPA axis, but new work has highlighted the need to investigate fear circuitry and neurogenesis. To improve on the current treatments and to overcome the challenges of developing novel therapies, further research is needed that focuses on elucidating the molecular mechanisms of PTSD, better understanding the pathophysiology, and establishing early diagnostic criteria. These goals can be achieved through rigorous pre-clinical research that will extend forward into clinical trials.

## Figures and Tables

**Table 1:** Genetic influences on PTSD.

Author	Population	Gene of Interest	Results
Amstadter et al. 2009	Hurricane Victims	RGS2 Polymorphism (rs4606)	Participants with the rs4606 polymorphism showed increased PTSD development and symptom severity in those exposed to a traumatic event with low social support.
Bachmann et al. 2005	Veterans (Vietnam)	Glucocorticoid Receptor (GR) Polymorphisms (N363S and BclI)	The frequency of GR polymorphisms were not increased in participants with PTSD. No changes in glucocorticoid sensitivity were observed in the PTSD group. The common GR polymorphisms observed in this study do not contribute to the risk of PTSD development.
Boscarino et al. 2012	Non-malignant chronic pain patients	FKBP5, COMT, CHRNA5, and CRHRI polymorphisms	Significantly higher rates of lifetime PTSD were observed in participants with SNPs in the four target genes: FKBP5 (rs9470080), COMT (rs4680), CHRNA5 (rs16969968), and CHRR1 (rs110402).
Bruenig et al. 2017	Veterans (Vietnam)	TNFα Polymorphism (rs1800629)	In a dominant model, significant associations were found between the TNFα rs1800629 polymorphism in the promotor region of the gene and the development of PTSD.
Dretsch et al. 2015	Veterans (Operation Iraqi Freedom/Operation Enduring Freedom)	APOE, DRD2, and BDNF Polymorphisms	A significant predictor of PTSD development was the BDNF Val66Met (rs6265) SNP. This BDNF polymorphism also correlated with significantly higher risk of incurring a mild TBI. There were no significant differences in PTSD (or TBI) frequency among any of the other observed genotypes.
Kimbrel et al. 2015	Veterans (Iraq/Afghanistan-Era)	Apolipoprotein E ε4 Allele (APOE ε4)	Significant effects were observed in non-Hispanic black veterans where APOE ε4 homozygotes exposed to high levels of combat experienced increased rates of PTSD, psychiatric comorbidity, and worse symptom severity when compared to APOE ε4 heterozygotes and non-carriers.
Logue et al. 2013	Veterans and their intimate partners	ANK3 Polymorphisms	There was a significant association with three ANK3 SNPs (rs28932171, rs11599164, and rs17208576) and the diagnosis of PTSD.
Voisey et al. 2009	Veterans (Vietnam)	DRD2 Polymorphisms (SNP C957T, deletion polymorphism −141delC) and DRD2/ANKK1 Polymorphisms (SNP Taq1A)	A significant increase in PTSD susceptibility was observed for the CC genotype of the C957T polymorphism. There was no significant association observed for the −141delC or Taq1A polymorphisms.
White et al. 2013	Hurricane Victims	CRHR1 Polymorphisms	A significant increased risk for developing PTSD symptoms was observed in carriers of the rs12938031 and rs4792887 CRHR1 polymorphisms. The rs12938031 polymorphism was also found to be significantly associated with PTSD diagnosis.
Winkler et al. 2017	Trauma patients experiencing an isolated and uncomplicated mild traumatic brain injury (mTBI).	COMT Polymorphism (rs4680)	The COMT Val158Met polymorphism (rs4680) is associated with increased frequency of PTSD and a poorer functional outcome following mTBI. The COMT Met158 allele is associated with lower PTSD frequency and improved functional outcome following mTBI.

**Table 2:** 24 h urinary cortisol and norepinephrine in participants by PTSD status.

24 hour urine	Never PTSD	Lifetime PTSD	Current PTSD	P-value
Cortisol (N:304/97/193)				
Mean(SD)	30.9 (21.1)	23.5 (14.6)	27.2 (20.2)	p=0.004 *
Log transformed	3.2 (0.7)	3.0 (0.7)	3.1 (0.7)	p=0.002 *
Epinephrine (N:314/100/199)				
Mean(SD)	3.9 (3.0)	3.4 (2.5)	4.1 (3.5)	p=0.18
log transformed	1.1 (0.8)	0.9 (0.8)	1.1 (0.8)	p=0.22
Norepinephrine (N:314/100/199)				
Mean(SD)	50.96 (24.2)	51.62 (28.3)	57.17 (27.9)	p=0.03 **
Log transformed	3.80 (0.6)	3.79 (0.6)	3.93 (0.5)	p=0.02 **
Dopamine (N:314/100/198)				
Mean(SD)	187.6 (99.2)	188.0 (104.1)	190.6 (95.0)	p=0.94
Log transformed	5.1 (0.6)	5.1 (0.6)	3.9 (0.6)	p=0.78
Urine creatinine over 24 h	1610 (504)	1566 (559)	1645 (548)	p=0.46
Serum creatinine (mg/dl)	1.0 (0.3)	1.0 (0.2)	1.1 (0.3)	p=0.22

Total: 613 participants, 199 (32.5%) had current PTSD, 100 (16.3%) had lifetime but not current PTSD, and 314 (51.2%) never had PTSD. The Table shows that a significant increase in cortisol and norepinephrine^17^.

* = denotes significance
